# Recent Trends in Designing Novel Foods: Nutritional Profiles and Consumer Perceptions

**DOI:** 10.3390/foods15050795

**Published:** 2026-02-24

**Authors:** Raquel P. F. Guiné, Maria João Barroca, Ofélia Anjos

**Affiliations:** 1 CERNAS-IPV, Polytechnic University of Viseu, Campus Politécnico, Repeses, 3404-510 Viseu, Portugal; 2 ESAC—Agriculture School of Coimbra, IPC—Polytechnic University of Coimbra, Bencanta, 3045-601 Coimbra, Portugal; mjbarroca@esac.pt; 3 CERNAS—IPCB, Polytechnic University of Castelo Branco, Quinta da Senhora de Mércules, 6001-909 Castelo Branco, Portugal; ofelia@ipcb.pt

## 1. Introduction

Designing novel foods is a pivotal issue for companies to remain active and competitive in today’s demanding food market. This design of novel food products entails significant challenges from diverse perspectives: technology, sustainability, food security, composition, nutritional profile, health effects, and modern market trends, to name a few.

The world population continues to rise while people’s standard of living is also improving. This results in an unceasing growing demand for food, exacerbating the pressure on food resources. Therefore, there is an urgent need to develop new food resources through a holistic, multifaceted approach [1].

Sustainability is a current underpinning of many new food development trends across several fundamental areas. In the area of sustainable packaging, the focus is on bio-based packaging [2], edible packaging [3], active [4] and intelligent packaging [5], or 3D printing packaging [6]. Also, there is a widespread emphasis on sustainable ingredients and valorisation of resources like food waste [7,8,9], sources of alternative and unconventional proteins [10], including animal proteins like edible insects [11] or plant-based proteins from fruits and vegetable by-products [12], algae [13], or mushrooms [14], for example.

The design of healthier foods, with improved composition and nutritional profiles that confer health benefits, has also been in the spotlight due to the increasing consumer demand for foods that not only satiate but also have positive health effects when consumed [15]. The challenges of improving food composition to achieve balanced nutritional profiles and greater richness in bioactive compounds have been addressed from many different perspectives. Fermented products [16], food design based on gut microbiota [17], functional foods [18] and nutraceuticals enhanced by nanotechnology [19] are some examples.

The unquestionable role of the consumer must not be seen only from the point of view of a future potential buyer, but also as an active element in the design of products throughout the development stages [20]. Consumer-based food design is, at present, a more reassuring approach for anticipating the success of the marketing phase of new foods. Some chefs and food designers have been developing collaborative activities together with academic professionals and sensory analysis experts [21]. Also, consumers have been integrating teams to develop alternative and innovative packaging solutions [22,23].

Research on novel food technology acceptance has seen significant advances in recent decades. The Unified Theory of Acceptance and Use of Technology (UTAUT) is undoubtedly one of the most comprehensive models to date. This model has advanced the Technology Acceptance Model (TAM) by assimilating insights from different theoretical approaches, enabling a better understanding of consumers’ acceptance and use of technology. The most relevant contributions comprise the incorporation of social influencing factors and facilitating circumstances [24].

## 2. Overview of Published Articles

This Special Issue gathered studies focusing on the development of new food products across all the domains this complex process involves, from conceptualisation to commercialisation and consumer acceptance.

The study by Nina G. Kon’kova and collaborators (Contribution 1) explores the potential of chufa tuber flour to enhance the functional properties of bread. They conducted diverse evaluations (biochemical, farinographic, and baking properties) on chufa tubers and bread samples with 30% replacement of wheat flour with chufa tuber flour. Their results revealed that most assessments improved with chufa tuber flour and bread containing chufa flour, compared with the control samples (whole-grain wheat flour and wheat bread). The improved biochemical indicators were oil content, fibre content, total phenolic compounds, and antioxidant activity, while no improvement was observed in protein and starch content. Regarding the technological properties, viscoamylography, farinographic sedimentation, and baking quality measurements confirmed that the bread dough incorporating chufa flour was stronger, more resistant to kneading, and showed a lower degree of liquefaction than the control. Also, a sensorial evaluation was conducted to compare the improved breads with the control samples. The authors concluded that chufa tuber powder enhances the rheological and sedimentation properties of bread dough, making it a possible ingredient to improve baking properties and the manufacture of functional food products.

The study by Hassan Barakat and collaborators (Contribution 2) evaluated the potential of sukkari date paste and mixed nuts as ingredients to improve the nutritional and biochemical profiles of high-energy bars. They compared five formulations with variable date-to-nut ratios ranging from 40:60 to 80:20. The results showed that higher percentages of date increased moisture and carbohydrates, whereas higher percentages of nut enhanced protein, fat, and energy. Regarding dietary minerals, they observed increases in calcium, phosphorus, magnesium, and trace elements as the percentage of date decreased. However, the phenolic compounds and antioxidant activity were highest in the bars with a higher percentage of nuts, and so were the ratio of total to essential amino acids and protein quality. Their findings evidence the potential of optimised date-nut bars as nutrient-dense functional snacks.

The study by Daniela Magalhães and collaborators (Contribution 3) is in line with the previous work and also explored the enrichment of a food, in this case, it was mortadella (a bologna-type sausage), through the addition of lemon powder rich in dietary fibre. The authors demonstrated that the functional lemon dietary fibre possessed antioxidant and antibacterial activities and reduced sodium levels and residual nitrite levels. Although the reduction in nitrites led to a slight increase in lipid oxidation, it remained below the rancidity threshold (≥1.0). The addition of lemon powder resulted in a paler colour and increased hardness, but the sensory evaluation received positive feedback.

The study by Rocio Lopez-Navarro and collaborators (Contribution 4) examined the consumer perceptions towards cashew nuts based on a neuroscientific and sensory approach, as a way to analyse both conscious and unconscious responses that allow understanding consumer preferences. Their results showed consumer preference for one of the analysed samples, which was confirmed by electroencephalogram metrics and frontal alpha asymmetry for flavour (showing greater activity in the left frontal lobe, associated with positive emotions, for the preferred sample). Task engagement measurements revealed higher engagement with the same sample during flavour evaluation. The authors believe that their findings have implications for product optimisation, market segmentation, and the development of marketing strategies to improve product commercialisation.

The study by Natália L. Seixas and collaborators (Contribution 5) examined the addition of fermented elderberries as a bread ingredient to enhance bread quality and biological value. They evaluated how incorporating fermented elderberries into a traditional bread affected its bioactivity and shelf life. They confirmed that fermentation increased the bioactive compounds and antioxidant activity of the elderberries, resulting in an extended shelf life for the studied bread.

The study by Pedro Coelho and collaborators (Contribution 6) investigated how a mixed system composed of white *Chlorella vulgaris* and a disruptive emulsifier (yeast protein extract), could be used to transform traditional coriander mayonnaise into an analogue product. The mixed system was found to confer stability to the mayonnaise, and the introduction of microalgae enhanced bioactivity, particularly total phenolic compounds and antioxidant activity. Sensorial evaluation confirmed the similarity of the products, except for the parameter colour.

The study by Alessandro Bianchiand and collaborators (Contribution 7) focused on enriching salty biscuits with bee pollen, in either fresh or dried form, to improve their functional characteristics. The authors studied the composition and sensory characteristics of the biscuits. Their results showed that biscuits with 5% fresh pollen had the best combination of chemical and sensory properties. They concluded that biscuits fortified with bee pollen can serve as a vehicle for the ingestion of phenolic compounds and carotenoids, while maintaining an acceptable sensory profile.

The study by Marcelina Maciejewska and collaborators (Contribution 8) analysed edible insect hydrolysates as sustainable sources of protein and investigated consumers’ attitudes towards insect-based foods in two European countries (Poland and Spain). Their approach was based on alignment between technical advancements and consumer preferences, to intertwine laboratory innovation with market feasibility, thereby contributing to the development of sustainable functional foods. They optimised the enzyme hydrolysis process to increase the antioxidant activity of the hydrolysates. On the other hand, consumer surveys confirmed that demographic and cultural characteristics significantly influence consumer acceptance of insect-based foods.

The last contribution (Contribution 9) is a review article by Hassan Muzaffar and collaborators that discusses the therapeutic potential of white kidney beans as a functional food. In their review, they examine the mechanistic roles of white kidney bean in weight regulation, though suppression of starch digestion, attenuation of postprandial glycaemia, modulation of appetite and satiety, and hypolipidemic effects. They concluded that white kidney bean has potential as a nutraceutical for metabolic health, supported by clinical evidence demonstrating its impact on reducing body fat mass, glycaemic excursions, and overall weight.

## 3. Final Remarks

The continuous interest in developing successful new food products that meet consumer expectations and needs has been the motor of change over the past decades, leading to innovative food products, new and improved ingredients, more sustainable and efficient production and transformation technologies, and new integrated food design approaches. 

The domain of novel foods development also continuously opens new doors to future work, leads to unforeseen approaches, and constitutes a field where technological advancement allied to multifaceted conceptualisation will undoubtedly have a positive impact in the future.

## Data Availability

Not applicable.
